# Management of *Mycoplasma genitalium* infections – can we hit a moving target?

**DOI:** 10.1186/s12879-015-1041-6

**Published:** 2015-08-19

**Authors:** Jørgen Skov Jensen, Catriona Bradshaw

**Affiliations:** Microbiology and Infection Control, Sexually Transmitted Bacterial Infections, Research and Development, Statens Serum Institut, Artillerivej 5, Copenhagen, DK-2300 Denmark; Central Clinical School. Monash University, Melbourne, VIC Australia; Melbourne Sexual Health Centre, The Alfred Hospital, Melbourne, VIC Australia

**Keywords:** *Mycoplasma genitalium*, Treatment, Antimicrobial resistance

## Abstract

*Mycoplasma genitalium* is an etiological agent of sexually transmitted infections, but due to its fastidious growth requirements, only a few *M. genitalium* strains are available for determination of the activity of currently used and new antimicrobial agents.

Recent clinical trials have demonstrated that treatment with azithromycin has decreasing efficacy due to an increasing prevalence of macrolide resistance, which is likely to be attributed to the widespread use of 1 g single dose azithromycin. Second line treatment with moxifloxacin is similarly under pressure from emerging resistance. The era of single dose monotherapy for uncomplicated STIs such as *M. genitalium* and *N. gonorrhoeae*, while convenient for patients and physicians, has been associated with escalating resistance and treatment failure and is now drawing to a close. There is a critical need for trials of combinations of existing registered drugs and new antimicrobial compounds, implementation of diagnostic testing combined with molecular detection of resistance, and antimicrobial surveillance.

## Review

### The importance of *M. genitalium* in genital syndromes

*Mycoplasma genitalium* was first isolated in 1980 from two of 13 men with non-gonococcal urethritis (NGU) [1]. It is an extremely slow-growing and fastidious bacterium, and progress in determining its role as a pathogen in human disease was hampered by the lack of reliable detection methods. After the development of the first diagnostic PCRs in the early 1990’s [2, 3], studies on male NGU started to accumulate [4, 5]. *M. genitalium* is now a well-established sexually transmitted infection and the etiological agent of a number of syndromes (reviewed in [6, 7]). Several studies have demonstrated the association between *M. genitalium* and urethritis, cervicitis, endometritis, and pelvic inflammatory disease (PID) [8–11]. In a recent meta-analysis [12], significant associations were found between *M. genitalium* and cervicitis (pooled odds ratio (OR) 1.66), and pelvic inflammatory disease (pooled OR 2.14). While there are less data in pregnancy, *M. genitalium* has been associated with preterm birth (pooled OR 1.89), and spontaneous abortion (pooled OR 1.82) [12], but the prevalence of *M. genitalium* in pregnant women has been low in some settings [13]. Serological studies and studies based on detection of *M. genitalium* using NAATs have also shown an association with increased risk of tubal factor infertility (pooled OR 2.43) [12]. In sub-analyses that accounted for co-infections, Lis et al. found these associations to be stronger and more statistically significant [12].

Several studies have demonstrated the association between *M. genitalium* and urethritis in men [4, 14–17] and in a meta-analysis including 37 studies up to 2010 [6], *M. genitalium* was associated with a pooled OR of 5.5 for NGU. In the 29 studies where information on chlamydial infection was available, *M. genitalium* was associated with a pooled OR of 7.6 for non-chlamydial non-gonococcal urethritis (NCNGU) [6]. The prevalence of *M. genitalium* in men with NCNGU ranges from 10 % to 35 % [6], thus contributing significantly to the overall burden of disease. In comparison, *M. genitalium* is detected in only 1 % to 3.3 % of men and women in the general Western European and United States population [18–20].

**Fig. 1:**
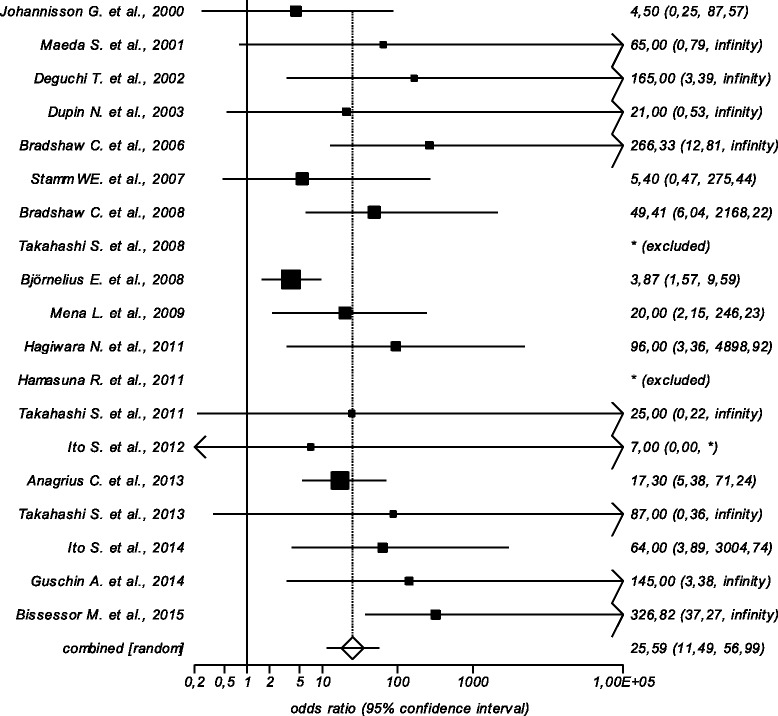
Meta-analysis of the risk of persistence of urethritis signs and/or symptoms in patients with and without eradication of *Mycoplasma genitalium.* Data from 19 studies included; in two studies, OR could not be calculated due to eradication of *M. genitalium* in all patients

Signs and/or symptoms of urethritis persist in patients in which antibiotic treatment fail. In men with persistent NCNGU after doxycycline therapy, as many as 41 % were found to be *M. genitalium* positive [21], and 91 % of patients with persistent *M. genitalium* infection experienced persistent urethral symptoms compared to 17 % of patients in whom *M. genitalium* was eradicated [22]. A total of 21 studies have examined the efficacy of treatment of *M. genitalium* positive urethritis, and presented data on the presence of urethritis in patients where antibiotic treatment failed to eradicate the infection [22–42]. Of the 310 patients with persistent *M. genitalium* infection, 240 (77 %) had persistent urethritis (defined as persistent urethral symptoms and/or signs). In the 19 studies where data on both men with persistent and eradicated *M. genitalium* infection could be evaluated, of the 285 patients with persistent *M. genitalium* infection, 220 (77 %) had persistent urethritis, compared to only 78 (16 %) of the 499 patients where *M. genitalium* was successfully eradicated (*p* < 0.0001). Analysing the 19 studies using random effects (DerSimonian-Laird method), persistent *M. genitalium* was associated with a pooled odds ratio of 26 (95 % CI = 11 to 57) for persistent urethritis (signs and/or symptoms). A forest plot illustrating the odds-ratios for the individual studies is shown in Fig. 1. Two studies reported no treatment failures, and ORs could therefore not be calculated [29, 33]. This analysis clearly shows that failure to eradicate *M. genitalium* leads to persistent or recurrent signs and symptoms of urethritis in a significant proportion of men with persistent infection.

### Antimicrobial treatment of *M. genitalium*

Similar to other mycoplasmas, *M. genitalium* lacks a rigid peptidoglycan containing cell wall [43] and, consequently, β-lactam antibiotics and other antibiotics targeting the cell wall are not active. Determining the spectrum of antimicrobial susceptibility in vitro has been hampered by the limited number of strains capable of growing well enough in mycoplasma broth or agar to enable determination of the minimal inhibitory concentration (MIC) by standard Clinical and Laboratory Standards (CLSI) approved methods [44]. However, it has been shown that using susceptibility testing based on antimicrobial growth inhibition of *M. genitalium* in Vero cell culture, provides similar results to those obtained by conventional methods [45, 46], and, consequently, larger collections including fastidious strains could be tested [47].

Early in vitro studies with few strains showed that *M. genitalium* was highly susceptible to tetracyclines and macrolides, particularly to azithromycin, but that it had reduced susceptibility to older quinolones such as ofloxacin and ciprofloxacin [48]. When more strains were studied, it became clear that some strains were resistant to the older quinolones with MIC90 (the MIC covering 90 % of the tested strains) for ciprofloxacin at 8 mg/l. However, all of these strains were susceptible to moxifloxacin and other quinolones with extended Gram-positive spectrum such as sitafloxacin, sparfloxacin, and gatifloxacin [46, 49]. With the emergence of resistance to both macrolides and quinolones as described below, the need for contemporary and representative strain collections has become evident in the search of new treatment modalities.

### Clinical efficacy of commonly used antimicrobials

#### Doxycycline

In vitro susceptibility testing suggests that most *M. genitalium* strains would be susceptible to doxycycline with a MIC_50_ of 0.25 mg/l and a MIC_90_ of 1 mg/l among 39 strains examined [47]. This is in striking contrast to clinical experience that predominantly showed poor efficacy of doxycycline in eradication of *M. genitalium* [50]. A single exception is an early study where 33 (94 %) of 35 men were *M. genitalium* negative after doxycycline treatment [51]. It should be noted, however, that eradication was evaluated only one week after treatment, and may reflect temporary suppression of the *M. genitalium* load, which has been reported in a study by Mena et al. [31] where 47 % of men with early clinical cure after doxycycline experienced a subsequent relapse. In controlled clinical trials, the microbiological cure rate has ranged between 22 % and 45 % [30, 31, 52, 53]. The reason for the discrepancy between in vitro and in vivo activity is not clear, but at least two studies [30, 38] have found that eradication rates in women were slightly higher than in men (37 % vs 17 %, and 48 % vs 38 %, respectively). Whether this is a reflection of lower compliance in men in relation to the nine-day doxycycline regimen used in these studies, or of an inaccessible prostatic focus of *M. genitalium,* remains speculative. However, based on these data doxycycline cannot be recommended for first line treatment of *M. genitalium* infection.

#### Azithromycin

In published studies, the majority of *M. genitalium* infected patients have been treated with azithromycin, and in early susceptibility studies [48], this macrolide was shown to be very potent. In patients with STIs, treatment with a 1 g single dose of azithromycin was well documented to be active in eradication of *C. trachomatis* [54], and the idea of a single-dose treatment was appealing for STI patients and widely adopted in many nations as first line treatment for NGU and *M. genitalium*. However, in *M. pneumoniae* pneumonia an extended treatment of 500 mg day one followed by 250 mg once daily on days two to five (referred to subsequently as extended azithromycin) was shown to be as effective as erythromycin for ten days [55]. Based on early, unpublished observations regarding the poor efficacy of doxycycline, Scandinavian researchers agreed that a slow-growing bacterium such as *M. genitalium* would need treatment for an extended period. Following the reported effect of extended azithromycin on the closely related *M. pneumoniae* [55], and approval of this regimen for treatment of pneumonia from the regulatory bodies, it was decided that this 5 day extended azithromycin regimen should be the preferred treatment for *M. genitalium* infections in Denmark, Norway and Sweden. A number of other extended regimens were considered, including giving a 1 g dose on day one in order to comply with treatment guidelines for *C. trachomatis,* and then supplementing with 250 mg on days two to five. This regimen may have increased the acceptance of the idea of extended treatment among physicians treating STIs, and would not be expected to have adverse influence on the treatment of *M. genitalium*. However, eradication of *C. trachomatis* infections with the 5 day extended azithromycin regimen has recently been documented [56].

Over the years, controversy has existed over the optimal dosage of azithromycin [57]. Importantly, no randomised controlled trial has compared azithromycin 1 g single dose with extended azithromycin, but a few observational studies and one treatment trial [30, 38, 58, 59] have included patients treated with both regimens. In the four studies, 469 *M. genitalium* infected patients received azithromycin as a 1 g single dose, and 244 received extended azithromycin with microbiological cure rates of 81 and 88 %, respectively (*p* = 0.026). It should be noted, however, that a large proportion of the patients receiving the extended azithromycin had it as a second line treatment, most often after doxycycline, impacting on direct comparison of the two azithromycin regimens. One of the emerging concerns with *M. genitalium* is that the 1 g single dose may be more likely to select for macrolide resistance compared to the extended regimen [57]. No data from controlled trials are available, and only a single observational study [38] has examined the development of resistance after extended azithromycin. This study found that none of 25 patients (0 % [95 % CI 0-13 %]) treated with extended azithromycin first line developed resistance, and, similarly, none (0 % [95 % CI 0-7 %]) of 52 patients who received extended azithromycin second line after doxycycline developed macrolide resistance. This contrasts with the risk of development of post-treatment resistance reported among 318 patients treated with a 1 g azithromycin in six studies, which was 10 % (95 % CI 7-14 %) [27, 38, 41, 60–62]. The 7 % difference in microbiological cure rate between 1 g single dose and extended azithromycin regimens calculated above is strikingly similar to the 10 % risk of development of post-treatment resistance after 1 g single dose azithromycin, lending support to the concept that single dose therapy appears to be associated with selection of resistance compared to extended regimens. The likely consequence of such rapid selection of resistant mutants without an efficient method to remove them from the population, is escalating macrolide resistance in high-risk populations. This situation appears to be playing out in countries where the use of 1 g azithromycin is widespread for chlamydia and its associated syndromes, and testing for *M. genitalium* is often restricted to speciality services or unavailable. Data from some of these nations, including in Greenland [63], Australia [60], Japan [64] and the UK [65], indicate macrolide resistance mutations are now detected in 30-100 % of cases with *M. genitalium*.

Macrolide resistance in *M. genitalium* is primarily caused by mutations in nucleotide 2058 or 2059 (*Escherichia coli* numbering) in region V of the 23S rRNA gene [66] although mutations in position 2062 have been described after treatment with josamycin in vivo [42] and in *M. pneumoniae* by in vitro selection [67]. Analogous to *M. pneumoniae*, it would be expected however, that strains with mutations in position 2062 would retain susceptibility to azithromycin. *M. genitalium* has only one ribosomal RNA operon, and as mycoplasmas have a high mutation rate [68], single nucleotide changes that confer high-level antimicrobial resistance would also be likely to be randomly present in a population of *M. genitalium* cells (heterotypic resistance). This could explain the recent observed association in three studies between a high organism load and increased risk of treatment failure following both azithromycin and josamycin [41, 42, 62]. Bissessor et al. reported in men with urethral *M. genitalium* that for each log10 increase in organism load there was a significant increase in the odds of 1 g azithromycin failure (adjusted OR, 1.8, *p* = .018) [41]. Macrolides are bacteriostatic, and it is therefore possible that a high initial organism load could lead to a larger number of organisms surviving the initial peak concentrations of azithromycin, with replication of the surviving cells ensuing when concentrations drop below the MIC leading to a situation where spontaneously occurring mutations would be readily selected. Overall, the body of evidence suggests that both heterotypic resistance with a minority population of resistant strains being present before initiation of treatment and resistance developing during treatment are likely to play a role in macrolide failure, with peak concentration and duration of antibiotic exposure, together with organism load, contributing to the survival and emergence of resistant mutants.

**Fig. 2:**
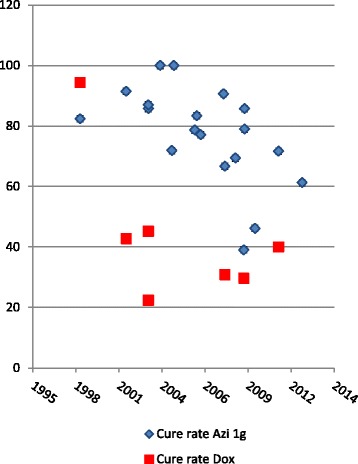
Microbiological cure rate of *M. genitalium* infections after azithromycin 1g single dose (blue diamonds) in 19 studies stratified according to the mid-date of the reported patient inclusion. In seven studies, data for cure rates after doxycycline treatment were available (red squares)

Cure rates for *M. genitalium* following 1 g azithromycin appear to be declining internationally, with evidence of a lower cure rate in studies where patients were recruited most recently. In Fig. 2, 19 studies were stratified according to the mid-date of the reported patient inclusion. In the seven studies recruiting patients in the first half of the observation period before December 2005 [10, 28–31, 51, 69], 222 (87 %) of 255 patients were microbiologically cured. In contrast, for the 12 studies with mid-enrolment after November 2005 [22, 32, 39, 41, 52, 53, 58–62, 70], only 660 (71 %) of 925 patients were cured (*p* < 0.0001). Seven of the studies also presented data on doxycycline treatment, and apart from the early study by Gambini et al. [51], cure rates were low, between 22 and 45 % (Fig. 2) but without an evident time trend.

#### Quinolones

Quinolones have been used in the treatment of *M. genitalium*, but based on in vitro susceptibility studies, it was predicted that second generation fluoroquinolones (ciprofloxacin and ofloxacin) would be inefficient in eradicating *M. genitalium* and this was confirmed in observational studies [23, 58] with an overall cure rate of 59 %. From in vitro data [46], the third generation quinolone levofloxacin appeared more promising, but in observational studies, again it only achieved a cure rate of 54 % in 82 treated patients [24, 34, 70, 71]

Moxifloxacin is a fourth generation quinolone and it has been one of the most commonly used second line antimicrobials being reported first in 2006 [27], although it had been used several years before. Moxifloxacin is bactericidal, and generally well tolerated, and in early studies, it appeared to have a cure rate approaching 100 % [22, 27, 38, 58]. However, warnings of possible hepatotoxicity were added to the US labelling in 2007, and in 2008, moxifloxacin was restricted to second line indications in Europe [72]. However, these serious adverse events were subsequently shown to be no more common in patients treated with moxifloxacin as monotherapy, than in patients treated with amoxicillin or with doxycycline [72]. Aside from concerns of adverse events, a declining cure rate for moxifloxacin has now been observed, primarily in patients from the Asia-Pacific region. Treatment failures have recently been reported in up to 30 % of patients treated with moxifloxacin, and a significant proportion of these patients’ strains had concurrent macrolide resistance mediating mutation leaving very few available treatment options [41, 59, 61, 70]. Resistance to moxifloxacin and other fourth generation quinolones is mediated by mutations in quinolone resistance determining regions (QRDR) of the *parC* gene, primarily in the hotspots in amino acid positions S83 and D87 (*M. genitalium* numbering) [73, 74] reported also in *M. pneumoniae, M. hominis* and *U. urealyticum* [75–77]. Based on data on in vitro selection of resistance in *M. pneumoniae*, supporting mutations in *gyrB* or *parE* may also be needed to reach resistant MIC levels [77], but this has not yet been definitely determined for *M. genitalium*. Mutations in the relevant positions have been found at a low rate in Europe with only 1 (5 %) of 22 samples from London, UK [65] and even lower than this in Denmark (Jensen, unpublished). In the Asia-Pacific region, however, evidence of fluoroquinolone mutations and associated treatment failure is emerging. In an Australian STI clinic 15 % of 143 specimens collected between 2008 and 2011, carried mutations that were associated with fluoroquinolone resistance [78]. Similarly, a report from Japan [64] found *parC* mutations in 17 (33 %) of 51 specimens collected between 2011 and 2013, with a dramatic increase from 20 % in 2011 to 47 % in 2013. It is important to note, however, that the in vitro and in vivo correlates of mutations in the QRDR for several of the reported mutations have not yet been established. The increasing trend of *parC* mutations reported from Japan was mainly due to an S83N mutation [64], and this substitution between two polar amino acids with uncharged side-chains has been shown not to change the moxifloxacin MIC from that of the susceptible wild-type G37 *M. genitalium* type-strain (Jensen, unpublished). This observation probably explains the remarkable 100 % cure-rate of sitafloxacin in nine *M. genitalium* infected patients with mutations in the QRDR of *parC*, eight of whom carried the indifferent S83N mutation.

Other fourth generation quinolones have been used in Japan. Gatifloxacin (now withdrawn from the market) cured 90 % of 48 patients [33, 71] and sitafloxacin has been used in five studies [36, 37, 39, 64, 70] with cure rates ranging from 100 % to 85 % with an overall cure rate of 95 % in 105 patients. In vitro, sitafloxacin also appear to have a good activity with MICs for moxifloxacin susceptible strains approximately one dilution step below that of moxifloxacin [46]. For moxifloxacin resistant strains, the MIC is currently being examined, but for one moxifloxacin resistant strain, an encouraging MIC of 1 mg/l has been found (R. Hamasuna, personal communication).

#### Other licensed but less commonly used antibiotics

##### Pristinamycin

Pristinamycin is an oral streptogramin antibiotic with bactericidal activity against Gram-positive organisms including methicillin-resistant *Staphylococcus aureus* (MRSA). It has a high activity against macrolide susceptible *M. genitalium* strains [48] and even for strains with combined macrolide and moxifloxacin resistance, the MIC remained <1 mg/l (Jensen, unpublished). Pristinamycin has been used for decades, particularly in France, for treatment of MRSA, but is not registered outside of France and some North-African countries. In *M. pneumoniae,* it has a relatively lower risk of development of resistance than azithromycin in vitro, and the selected mutants retained an azithromycin susceptible phenotype [67]. How a strain with pre-existing azithromycin resistance would react to selection with pristinamycin remains unknown, but in vitro selection studies are currently ongoing. Pristinamycin has been successfully used as third line treatment for patients infected with multidrug resistant *M. genitalium* strains. As most of these patients were facing their last known active antimicrobial therapy, the maximal recommended dose of 1 g four times orally a day for 10 days was used. Several patients in Scandinavia have responded well to this treatment, and recently a case series from Australia demonstrated six cases with successful eradication [41]. Using pristinamycin as a second line antibiotic instead of moxifloxacin would probably be hampered both by the price of pristinamycin and of the compliance issues associated with four times a day dosage. Whether lower daily doses would be effective remains to be determined, but for patients failing azithromycin, moxifloxacin, and possibly 14 days of doxycycline, a dose reduction is not advisable due to the proportion of these multidrug resistant strains that have an elevated MIC of 0.5 mg/l (Jensen, unpublished).

### Antimicrobials under development

Solithromycin is a newly developed fluoroketolide (CEM-101), a further development of the macrolides. It has a potent activity against azithromycin susceptible *M. genitalium* strains and to some extent also against azithromycin resistant strains [47]. Susceptibility to solithromycin appears to differ according to the type of macrolide resistance mediating mutation present, with eight strains harbouring mutations in position A2059 having susceptible MICs <2 mg/l, but only two of five strains with the A2058G mutation with susceptible MICs [47]. Using a clinical scenario seen in Denmark where 60 % of azithromycin resistant strains carry the A2058G mutation [79], one would expect to achieve a 65 % cure rate with the use of solithromycin for azithromycin resistant strains [47]. Treatment trials of single dose solithromycin for patients with gonorrhoea are currently underway, and in one study, a few cases of concomitant *M. genitalium* infections have been detected of which some have been eradicated [80]; however, data on the efficacy of solithromycin for *M. genitalium* is limited. Treatment trials targeting patients with NGU are likely to need a longer treatment duration in order to cover *M. genitalium* infections.

Lefamulin (BC-3781) belongs to the pleuromutilin class of antimicrobials, which has been used for decades in the veterinary industry for infections in pigs, and to a lesser extent in poultry, but this class has not previously been developed for human use. Lefamulin has a very potent activity in vitro with MIC ≤ 0.06 mg/l against multidrug resistant *M. genitalium* strains [81]. In the veterinary mycoplasmas, mutations in position A2058 have been associated with increased MIC levels, but only in a background of other mutations in the 23S rRNA gene, suggesting multistep development of significant resistance [82]. Lefamulin has successfully completed phase II trials for skin and soft tissue infections [83] and is currently entering trials for community-acquired pneumonia. It has not been studied in the treatment of STIs or specifically *M. genitalium*.

A novel spiropyrimidinetrione AZD0914 (a DNA gyrase inhibitor) has been tested against azithromycin and quinolone susceptible strains of *M. genitalium* and MIC <1 mg/l was reported for 11 strains [84]. No reports on human use of this interesting class of compounds have been published.

LBM415 belongs to a new antimicrobial class, the peptide deformylase inhibitors which inhibit bacterial protein synthesis. The compound has high in vitro activity against *M. pneumoniae* and was efficient in eradicating the bacterium in a mouse model [85], but unfortunately, prolonged high dosage produced methaemoglobulinaemia [86] and further development of the compound has apparently been stopped.

### Possible use of other registered antimicrobials

Although tetracyclines appear to be active in vitro, they have never shown evidence of sufficient efficacy clinically. Whether newer modifications of the tetracyclines such as the glycylcycline tigecycline would be active in *M. genitalium* infections remain to be determined. This antibiotic is active in vitro against *M. pneumoniae* [87], but at present, no in vitro data are available for *M. genitalium*, and the need for parenteral administration would not render it an optimal treatment, except in otherwise untreatable infections. Linezolid with its Gram-positive spectrum does not appear promising according to the MICs obtained for *M. pneumoniae* [87], but it has not been evaluated for *M. genitalium.*

Chloramphenicol has a relatively high MIC in vitro (5-25 mg/ml) [49] and although organisms with an MIC ≤16 mg/l are considered susceptible, is not likely to be a promising alternative. A derivative of chloramphenicol, thiamphenicol, has been used in treatment of NGU [88] and PID [89], and is reported to have a higher activity than chloramphenicol against mycoplasmas. It is currently registered in Italy and Brazil. Spectinomycin is widely used in the veterinary industry to treat mycoplasmal infections, however, most often in combination with lincomycin, probably to reduce the rapid development of resistance and due to a synergistic effect. The spectinomycin MIC breakpoint for *N. gonorrhoeae* is 32-64 mg/l and the MIC for *M. genitalium* has been reported to be <25 mg/l [49]. Since spectinomycin is inactive against *C. trachomatis* but has some activity against ureaplasmas, it has been used in the past to substantiate the role of ureaplasmas in NGU [90]. Spectinomycin may have a potential role as a treatment option for multidrug resistant strains of *M. genitalium* but the need for parenteral administration would be problematic. Lastly, successful eradication of ureaplasmas has been reported in a hypogammaglobulinaemic patient with untreatable urethritis after treatment with netilmicin [91]. However, aminoglycosides would not be expected to be active in vivo against *M. genitalium*, as even for the most potent aminoglycoside, netilmicin, the MIC of some *M. genitalium* strains was 25 mg/l [49]. Furthermore, as *M. genitalium* is capable of surviving intracellularly [92, 93], aminoglycosides would not be optimal as their intracellular concentration is very low.

## Conclusions

*M. genitalium* has shown a remarkable capability to develop antimicrobial resistance very rapidly after introduction of new treatment modalities. It has already become a difficult bacterium to treat on a syndromic basis, and even after specific detection, enormous local differences exist in the prevalence of strains with macrolide resistance-mediating mutations, highlighting the need for widespread antimicrobial resistance surveillance. In the ideal clinical setting, specific diagnostic tests for *M. genitalium* would be as readily available as tests for *C. trachomatis* and *N. gonorrhoeae,* and detection of *M. genitalium* would be accompanied or followed by molecular detection of macrolide resistance mediating mutations. For patients infected with a macrolide susceptible strain, evidence suggests that extended azithromycin should be the first-line treatment, but a test of cure should be routinely performed at three to four weeks, as resistance may develop even with the extended regimen. Patients with macrolide resistance can in most settings still be treated with moxifloxacin 400 mg once daily for 7-10 days, but treatment failure can occur, particularly in the Asia-Pacific region, where pre-existing quinolone resistance is common and, therefore, a test of cure 3-4 weeks after treatment is again highly recommended. If a patient experiences failure of azithromycin and moxifloxacin in the absence of reinfection, whether doxycycline 100 mg twice daily for 14 days should be attempted before pristinamycin is not clear, but the former is more readily available in most clinical settings. Most patients responding to doxycycline will have a negative test of cure by the end of treatment [94], but if they are still positive, treatment with pristinamycin 1 g four times daily for 10 days is currently the only third or fourth line antimicrobial treatment that is available. The important question is what to do when this treatment fails, which has been experienced by a limited number of patients but will inevitably increase in the foreseeable future. Based on the rapid emergence of resistance to first and second line treatment options internationally, continuous monitoring of the efficacy of treatment regimens for *M. genitalium* is highly recommended in clinical settings. In patients failing treatment, samples appropriate for culture should ideally be collected in order both to isolate *M. genitalium* for confirmation of the in-vitro correlates of antimicrobial resistance, and to elucidate the molecular mechanisms leading to treatment failure.

In clinical settings where access to testing for *M. genitalium* is not currently available, consideration should be given to changing presumptive treatment of NGU and cervicitis from the current recommendation of 1 g azithromycin to doxycycline in order to minimise the development and escalation of macrolide resistance. Patients failing doxycycline could then be subsequently treated with extended azithromycin, or in settings where macrolide resistance is already widespread, with moxifloxacin, if available. Access to *M. genitalium* testing in patients failing secondary treatment is available in many countries or via international collaborations, and should be used in these specific cases in order to optimise patient management.

Future research should focus on in vitro evaluation and subsequent treatment trials of untested, but registered antimicrobials, and emphasis should be on the development of treatment algorithms including dual therapy and resistance testing in order to minimise the development of resistance. Such combination therapies will need to be rapidly adopted and accompanied by antimicrobial resistance surveillance in order to retain susceptibility. As it has been the case for *N. gonorrhoeae*, the rapid development of resistance in *M. genitalium* highlights the fact that the era of single dose therapy for uncomplicated STIs is long past. Combination therapy and resistance testing together with the development and evaluation of new classes of antimicrobials should enable us to finally hit this fast moving target.
